# Editorial: Interrelationships between human microbiology and health of the elderly

**DOI:** 10.3389/fcimb.2024.1345103

**Published:** 2024-01-23

**Authors:** Xu Jia

**Affiliations:** Non-coding RNA and Drug Discovery Key Laboratory of Sichuan Province, School of Basic Medical Sciences, Chengdu Medical College, Chengdu, China

**Keywords:** drug resistance mechanism, gut microbiota, viruses, bacteria, aging

This Research Topic focuses on the relationship between human microbes and the health of older people, and the research content of the article is as follows:

When SARS-Cov-2 infects human beings, the virus spreads quickly, and there is no specific treatment drug. It also faces the severe challenge of co-infection and repeated infection of bacteria and viruses. Pathogenic bacteria are one of the major threats that increase the risk of complications and death in COVID-19 patients, and attention should be paid to the rational use and effective control of antibiotics during treatment. Gao et al. investigated and analyzed the main types of respiratory pathogens that infected COVID-19 patients, providing important reference values for the clinical treatment of COVID-19 infection.

With the emergence of SARS-CoV-2 mutants, especially the prevalence of Omicron mutants, it continues to evolve to enhance immune evasion, posing serious challenges to clinical treatment. All current therapeutic monoclonal antibodies are ineffective against BQ.1 and XBB infections. Older people, especially those with basic conditions such as high blood pressure, are at increased risk of death from the virus even after vaccination. Therefore, small molecule drugs targeting conserved targets are one of the extremely effective treatments. The results show that the Nsp1 target is very conservative among the mutant strain and the original strain. There is no mutation at the key amino acids, so Nsp1 can be used as a drug target, and the treatment against Nsp1 will target all variants of SARS-Cov-2 (Shen et al.).

The study focused on the effects of viral and bacterial infections on aging and immune senescence. Although the link between pathogens and aging has been reported, the underlying molecular mechanisms associated with them remain unclear, and whether infection induces aging remains to be studied. In this Research Topic, Gaglani et al. analyzed the reports of age-related biomarkers and cell markers in immune and non-immune cells at the time of infection. It was found that in the treatment of tuberculosis caused by mycobacterium tuberculosis, the bactericidal effect of vitamin C on mycobacterium tuberculosis *in vitro* was mainly manifested as increasing iron content, increasing reactive oxygen species (ROS) production, and increasing DNA damage. In addition, VC has significant multiple effects in the process of protein folding companion, information pathway, metabolic regulation, and other biological activities. The authors reviewed the role of vitamin C in the treatment of mycobacterium tuberculosis infection, clarified the generation of free radicals and the bactericidal mechanism of existing treatment schemes, and proposed the scheme of using ROS production as a drug research and development target to develop clinical drugs further.

The authors investigated changes in the gut microbiota and the effects of intravenous antibiotic therapy before and after heart surgery in elderly patients. It was found that after intravenous antibiotic treatment, the diversity of intestinal flora increased, and the composition of intestinal flora changed significantly. Zunyimycin C inhibited autophagy and promoted cell survival by activating the PI3K-AKT-mTOR pathway, mediated reactive microglia, and played a neuroprotective role in reactive astrocytes. Decreased the secretion of IL-1β and IL-6 in brain tissue, thus alleviating neuroinflammation in AD mice, achieving the effect of improving learning and memory impairment. It is speculated that Zunyimycin C plays an immune-enhancing role by changing intestinal flora diversity and SCFAS (Wang et al.).

In the face of Alzheimer’s disease in elderly patients, Zheng et al. used network pharmacology to search the analytical database for bioactivity, potential therapeutic targets, and AD-related therapeutic targets of Huanglian jiedu decoction. Through bioinformatics, including protein-protein interactions, the KEGG, analysis, and gene ontology (GO), major bioactive components, potential therapeutic targets, and key signaling pathways were obtained. The possible binding modes of the active compound to the core target were predicted by molecular docking. The results indicate that HLJDD may regulate the homeostasis of human flora through multi-target and multi-pathway so as to achieve the effect of treating Alzheimer’s disease.

Reyes et al. focused on the effects of intravenous antibiotics on the gut flora and methylnaphthoquinone biosynthesis in patients, especially elderly cardiac surgery patients. Surgical site infection is a common complication after surgery, and antibiotics are used to prevent or treat infection in the perioperative period. The study collected 388 stool samples from 154 heart surgery patients. The V3-V4 hypervariable region of 16S rRNA gene was amplified and then analyzed by sequencing. α- and β-diversity was analyzed at the OTU level and then classified according to the antibiotic combination. PICRUSt2 method was used to predict the effect of intestinal microbiota on methylnaphthoquinone biosynthesis. The findings suggest that intravenous administration of antibiotics is the main cause of disruption of gut microbiota composition in elderly heart surgery patients. After more than a week of antibiotic treatment, antibiotics began to have an effect on the gut microbiota, resulting in significant changes in the composition of the gut microbiota, increasing the richness and diversity of the microbiota. If the intravenous injection is stopped for more than two weeks, the gut microbiota will return to the state before the use of antibiotics. Sex was not a factor in the composition of intestinal flora. Cefoperazone sulbactam is used for a long time, which will affect the blood clotting function of patients.

Cell senescence is an important and irreversible biological process. The cellular process of aging may have both beneficial and harmful effects on the individual organism, it can play a dual regulatory role in the process of the body. On the one hand, it can be used as an effective anti-cancer mechanism and reduce the symptoms of some diseases, such as atherosclerosis, liver fibrosis and kidney fibrosis. The accumulation of senescent cells creates an environment of pro-inflammatory substances that negatively affect the tissue function of cells and promote the development of aging-related diseases. For example, the accumulation of senescent cells is associated with pulmonary fibrosis and type 2 diabetes, which damages the body’s immune system and reduces the body’s ability to respond to infection, leading to autoimmune diseases. Therefore, regulating aging by balancing aging and non-aging processes may have good therapeutic effects. However, how to maintain the homeostasis and health state in the cell needs to be studied and confirmed in the case of pathogen infection. Although an association between aging and pathogens has been reported, the definitive link between the two and the associated molecular mechanisms remain to be studied and confirmed. Xue et al. review and discuss recent reports describing cellular markers and biomarkers in an attempt to better understand the relationship between both aging and pathogens. In addition, some pathogens regulate aging for their own benefit, have evolved relevant molecular mechanisms to induce aging, and use this cellular process of aging to complete the replication cycle. But the immune system, already in a state of immunosenescence, may also allow latent viruses to be reactivated more frequently, leading to a new round of infection in the host. Therefore, by regulating this senescent cell state, it will become a good therapeutic target for the treatment of some specific infectious diseases in the future.

## Author contributions

XJ: Writing – original draft, Writing – review & editing.

